# The Differential Diagnosis of Disease of the Sound-Conducting and of the Sound-Perceiving Apparatus

**Published:** 1887-06

**Authors:** 


THE DIFFERENTIAL DIAGNOSIS OF DISEASE OF THE SOUND-CONDUCTING AND OF THE SOUND-PERCEIVING APPARATUS.

Bartsch, of Parchim, in the *Archives of Otology*, volume XV., numbers 2 and 3, recommends an experiment which seems to him more serviceable then Gellé's experiment in the differential diagnosis above mentioned. It is thus condensed by Dr. C. S. Bull, in the *New York Medical Journal:* The experiment is based on the attempted exclusion from the auditory function of the sound-conducting portion, by rarefaction of the air in the external auditory canal. The air being exhausted from the meatus by means of a rubber tube hermetically inserted into it, the drum-head will be rendered to some extent incapable, at least during great negative pressure, of propagating to the labyrinth the vibrations which impinge upon it. Under great preasure thus exerted upon one's self, one feels how the drum-head is extended outward, and, since with such great tension of the drum-head the malleus is also withdrawn from the incudal joint, the transmission of the sound-waves by the drum-head and the ossicles will be thus greatly hindered. In that case, therefore, only those vibrations will be conducted to the labyrinth which are directly propagated by the bones which do not touch the drum-head—*i. e.*, the cranial vibrations. The question then will be, how intensely and how long are these vibrations preceived? Are they of the same intensity, are they perceived for the same length of time as when the tuning-fork vibrates on the head without the air being exhausted from the meatus? In that event, we must assume that the sound-conducting apparatus performs its function badly. Or else, if they are much fainter, or are heard for a much shorter time than before, the sound-conducting apparatus is healthy. The experiment proves that direct bone-conduction really produces auditory sensations.

